# Impact of continued administration of tolvaptan on cirrhotic patients with ascites

**DOI:** 10.1186/s40360-018-0277-3

**Published:** 2018-12-18

**Authors:** Tomomi Kogiso, Takaomi Sagawa, Kazuhisa Kodama, Makiko Taniai, Katsutoshi Tokushige

**Affiliations:** Department of Internal Medicine, Institute of Gastroenterology, Tokyo Women’s Medical University, 8-1 Kawada-cho, Shinjuku-ku, Tokyo 162-8666 Japan

**Keywords:** Vasopressin V2-receptor antagonist, Tolvaptan, Liver cirrhosis, Body weight reduction, Long-term outcome

## Abstract

**Background:**

The vasopressin V2-receptor antagonist tolvaptan is used to treat cirrhotic patients with ascites. We investigated the outcome of long-term treatment.

**Methods:**

This was a single-center retrospective study. Overall, 170 cirrhotic patients (95 males, median age 63 years) were enrolled and received tolvaptan orally after hospitalization for ascites, which included treatment with conventional diuretics. We compared patients who withdrew tolvaptan treatment after < 1 year (*n* = 90) with patients who continued treatment for ≥1 year (*n* = 37). In continuously treated patients, the pretreatment and post-treatment (1 year) blood biochemistry values were assessed.

**Results:**

Overall, 37 patients received treatment for ≥1 year and showed a higher response after tolvaptan therapy. The reduction in body weight was 2.0 (− 3.4–17.2) kg compared to discontinued cases, which had a body weight reduction of 1.1 (− 6.2–7.5) kg after 1 week. The group that received treatment for ≥1 year had a significantly lower rate of the complication gastroesophageal varices and also showed better liver function. In patients with continued treatment, serum levels of albumin was significantly higher without renal disturbance after 1 year of treatment. The prothrombin time/international normalized ratio and ammonia level were also significantly improved. Multivariate analyses showed that a change in body weight reduction and serum levels of albumin were predictive factors of continued administration.

**Conclusions:**

Long-term tolvaptan treatment increased serum levels of albumin, decreased ammonia levels, and preserved renal function after 1 year of treatment. A reduction in body weight after 1 week was associated with a favorable outcome of tolvaptan therapy.

## Background

Liver cirrhosis occurs in end-stage liver disease, and ascites accumulation is a sign of decompensated liver cirrhosis. Following the development of ascites, the 5-year survival rate is only 50% [1, 2]. After the development of dilutional hyponatremia, refractory ascites, and type 2 hepatorenal syndrome, the 1-year probability of survival is 25.6, 31.6, and 38.5%, respectively [2]. Ascites treatment for liver cirrhosis patients is important, and the vasopressin V2-receptor antagonist tolvaptan has dramatically improved this treatment [3, 4]. The Japanese Society of Gastroenterology recommended the use of tolvaptan prior to albumin administration or ascites drainage in 2015 [5]. Arginine vasopressin (AVP) triggers vasoconstriction by binding to V1a receptors, and promotes water re-absorption in the kidney by binding to V2 receptors, which are primarily responsible for the antidiuretic effects of AVP [3]. Tolvaptan selectively blocks the binding of vasopressin to V2 receptors.

In previous studies, we have shown that treatment with tolvaptan for 6 months is efficacious and safe and may improve the prognosis of cirrhotic patients [6, 7]. Median survival is significantly longer in patients with less ascites accompanying diuretic treatment [8]. According to a meta-analysis of randomized controlled trials, a tolvaptan-associated reduction in diuretic use is associated with lower serum sodium levels and maintenance of renal function, but no survival benefits were apparent in either the short- or long-term [9].

Tolvaptan response does not depend on albumin or sodium levels [3, 10]. These characteristics are beneficial for cirrhotic patients because cirrhosis results in extremely low levels of albumin and sodium in end-stage liver disease [11]. Conventional diuretics, particularly furosemide, bind reversibly to carrier protein; therefore, their mechanism of action requires albumin as the carrier protein. This leads to a decrease in the action of diuretics in cirrhosis. In addition, cirrhosis is often complicated by hyponatremia. It is sometimes difficult to treat using conventional diuretics because reducing or abolishing sodium chloride reabsorption further reduces serum levels of sodium, and hyponatremia is also associated with poor prognosis [12, 13]. Tolvaptan increases sodium levels and improves survival [13].

However, no studies have investigated long-term tolvaptan administration. Here, we investigated whether long-term tolvaptan treatment may improve liver and renal function, the predictive factors of long-term treatment, and the characteristics of patients who could stop treatment after improvement of ascites.

## Methods

### Patients and study design

**Fig. 1:**
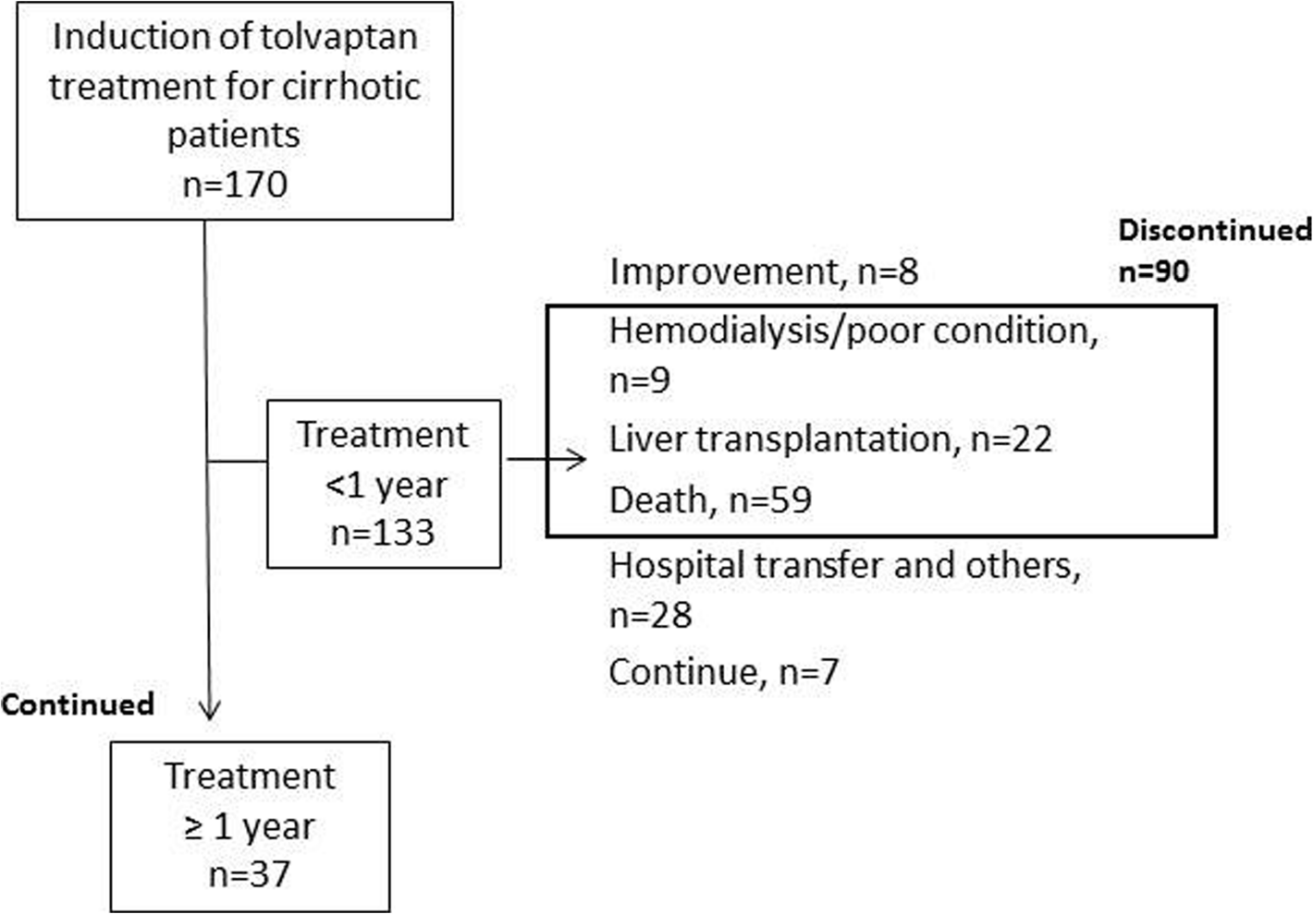
Flow chart of the study design. Overall, 170 cases with cirrhosis were treated with tolvaptan. Eight patients finished treatment due to improved ascites, and treatment was stopped in 90 cases due to poor condition, death, and liver transplantation. By contrast, 37 cases were continuously administered tolvaptan for ≥1 year

This was a single-center, retrospective, observational study conducted between September 2013 and July 2018. A total of 170 cirrhotic patients (95 males, 56%, 75 females, 44%), complicated with ascites, who received tolvaptan (Samsca™; Otsuka Pharmaceutical Co. Ltd., Tokyo, Japan) after hospitalization with a salt/water-restricted diet (including four retreatment patients) were enrolled. Tolvaptan was started from a half dose (3.75 mg once per day) and it was increased to 7.5 mg/day if the response or body weight reduction was insufficient. Tolvaptan was added on other diuretics including 0–160 mg/day furosemide and/or 0–400 mg/day spironolactone. Excluded criteria was patients with severe renal dysfunction (estimated glomerular filtration rate [eGFR] < 15 mL/min/1.73 m^2^ or serum levels of creatinine > 3.5 mg/dL) and patients with a hepatic coma scale score > II. Overall, of the patients who were excluded in the discontinued cases (*n* = 90), eight patients could stop treatment and seven continued treatment for < 1 year. Patients who received tolvaptan treatment for < 1 year and ≥ 1 year (*n* = 37) were compared (Fig. 1). Denver shunts were placed following the manual of the procedure [14].

This study was conducted according to the principles of the Declaration of Helsinki and the ethics rules of Tokyo Women’s Medical University Hospital (TWMU, Tokyo, Japan). The TWMU Institutional Review Board approved the study protocol (approval no. 3258-R).

### Clinical parameters

The following baseline characteristics of patients were assessed: age, sex, clinical history, body weight, urine volume, underlying hepatic diseases, rates of complications of cirrhosis (i.e., varices, hepatocellular carcinoma, and hepatic encephalopathy), treatments including the administration of diuretics and branched-chain amino acids (BCAA), and procedures including ascites drainage and implantation of Denver shunts. Blood samples for biochemistry and hematological data were collected at the time of administration of tolvaptan and after 1 year of treatment. Urine volume was determined the next day and the reduction in body weight was evaluated after 1 week. Laboratory tests included serum concentrations of albumin, total bilirubin, aspartate aminotransferase, alanine transaminase, γ-glutamyltransferase, ammonia, alpha-fetoprotein and des-gamma-carboxy prothrombin, platelet counts, hematocrit, prothrombin time (PT), and PT/international normalized ratio (INR). The modified Child-–Pugh (CP) score [15] and the model for end-stage liver disease (MELD) score [16] were used to evaluate liver function.

### Follow-up and outcomes

The patients were hospitalized and administered tolvaptan for 1–2 weeks. After discharge, patients were followed every 1–2 months at an outpatient clinic to check biochemical parameters. One year after tolvaptan administration, outcomes were monitored and evaluated the efficacy and the condition.

### Statistical analyses

Data are presented as medians with minimum and maximum values. Significant differences between the two groups were assessed using the Mann–Whitney U test and χ^2^ test with the SPSS statistical software package (SPSS Inc., Chicago, IL, USA). Pretreatment and post-treatment values were compared using Wilcoxon signed-rank tests. Differences were considered statistically significant at *p* < 0.05. Multivariate logistic regression analyses were performed with the likelihood ratio test to assess their fit after 1 year of tolvaptan treatment.

## Results

### Baseline characteristics of patients pretreated with tolvaptan

A flow chart of our study is shown in Fig. 1. A total of 170 cirrhotic patients were treated with tolvaptan and 64% of patients had their dose increased to 7.5 mg/day. The median age was 63 (range, 21–90) years old, and 56% were male (Table 1). Underlying liver diseases included hepatitis C virus (HCV, 28%), hepatitis B virus (HBV, 7%), alcoholic liver disease (ALD, 28%), non-alcoholic fatty liver disease (NAFLD, 11%), and primary biliary cholangitis (PBC; 10%). Regarding complications of liver cirrhosis, gastroesophageal varices (68%), hepatocellular carcinoma (HCC; 32%), and hepatic encephalopathy (27%) were observed.

**Table 1:** Baseline characteristics of the patients and laboratory data at initiation of tolvaptan

	Total (*n* = 170)	Discontinued* (*n* = 90)	Continued** (*n* = 37)	*p*-value * vs. **
Age (years)	63 (21–90)	60 (21–85)	63 (22–90)	0.28
Sex (% of males)	95/170 (56%)	47/90 (52%)	23/37 (62%)	0.31
BW (kg)	61.9 (34.4–143.6)	61.6 (37.4–98.9)	62.9 (34.4–143.6)	0.48
BW after 1 week (kg)	60.9 (32.7–136.2)	61.5 (36.3–103.8)	61.0 (32.7–136.2)	0.75
BW reduction after 1 week (kg)	1.5 (−6.2–17.2)	1.1 (−6.2–7.5)	2.0 (−3.4–17.2)	0.03
Urine volume (mL)	1595 (120–6630)	1450 (120–6630)	1690 (195–4460)	0.77
Etiology of liver disease (%)				0.07
HCV	48 (28%)	28 (31%)	6 (16%)	
HBV	12 (7%)	4 (4%)	4 (11%)	
ALD	48 (28%)	22 (24%)	15 (41%)	
NAFLD	19 (11%)	7 (8%)	5 (14%)	
PBC	17 (10%)	7 (8%)	4 (11%)	
Others	31 (18%)	24 (27%)	4 (11%)	
Complication (%)				
Gastroesophageal varices	109/160 (68%)	62/82 (76%)	21/37 (57%)	0.04
Hepatocellular carcinoma	54/170 (32%)	26/90 (29%)	13/37 (35%)	0.51
Hepatic encephalopathy	46/170 (27%)	33/90 (37%)	8/37 (22%)	0.10
Diuretics				
Furosemide dose (mg/day)	20 (0–160)	20 (0–120)	20 (0–80)	0.48
Spironolactone dose (mg/day)	50 (0–400)	50 (0–400)	50 (0–150)	0.24
Branched-chain amino acid (%)	150/170 (88%)	76/90 (84%)	35/37 (95%)	0.12
CART or drainage (%)	84/170 (49%)	47/90 (52%)	15/37 (41%)	0.37
Denver shunt	6 (4%)	1 (1%)	3 (8%)	0.04
Laboratory data				
Albumin (g/dL)	2.4 (1.5–4.2)	2.4 (1.6–3.6)	2.6 (1.7–4.2)	0.03
Total bilirubin (mg/dL)	1.9 (0.3–52.4)	2.2 (0.3–52.4)	2.0 (0.4–21.7)	< 0.01
Aspartate aminotransferase (U/L)	48 (10–551)	52 (12–551)	42 (13–171)	0.06
Alanine aminotransferase (U/L)	28 (3–381)	29 (3–381)	29 (7–144)	0.53
γ-Glutamyl transpeptidase (U/L)	49 (9–269)	52 (9–269)	48 (9–229)	0.28
Platelet count (×10^4^/μL)	8.8 (1.5–62.7)	8.8 (2.1–62.7)	7.6 (1.5–23.9)	0.10
Prothrombin time (PT%)	55.6 (16.3–90.3)	52.5 (16.3–89.0)	54.5 (19.5–90.3)	0.22
PT/INR	1.31 (0.98–3.17)	1.33 (1.04–3.17)	1.32 (0.98–2.84)	0.36
Ammonia (μg/dL)	66 (16–269)	60 (24–212)	85 (25–269)	0.11
α-fetoprotein (ng/mL)	4 (1–142,010)	4 (1–142,010)	4 (1–210)	0.13
Des-gamma-carboxy (mAU/mL)	68 (3–357,790)	73 (5–357,790)	27 (3–4994)	0.26
Blood urea nitrogen (mg/dL)	20.9 (5.5–125.3)	22.5 (5.8–64.3)	23.2 (6.0–63.3)	0.86
Creatinine (mg/dL)	0.99 (0.20–3.30)	1.00 (0.20–2.87)	1.04 (0.44–3.30)	0.28
eGFR (mL/min/1.73 m^2^)	53.9 (14.9–250.6)	53.1 (14.9–250.6)	49.5 (16.8–107.2)	0.06
Serum sodium (mEq/L)	136 (118–145)	135 (118–143)	137 (122–145)	0.01
Modified Child-Pugh (CP) score	11 (6–14)	11 (8–14)	11 (8–13)	0.05
MELD score	12 (1–37)	14 (1–37)	12 (2–29)	0.53

*n* number of patients, *BW* body weight, *HCV* hepatitis C virus, *HBV* hepatitis B virus, *ALD* alcoholic liver disease, *NAFLD* non-alcoholic fatty liver disease, *PBC* primary biliary cholangitis, *CART* cell-free and concentrated ascites reinfusion therapy, *eGFR* estimated glomerular filtration rate, *INR* international normalized ratio, *MELD* model for end-stage liver disease. Data are represented as the median (range)

The median urine volume on the next day of treatment was 1595 (120–6630) mL and the body weight change was − 1.5 (− 17.2 to + 6.2) kg after 1 week of treatment.

**Fig. 2:**
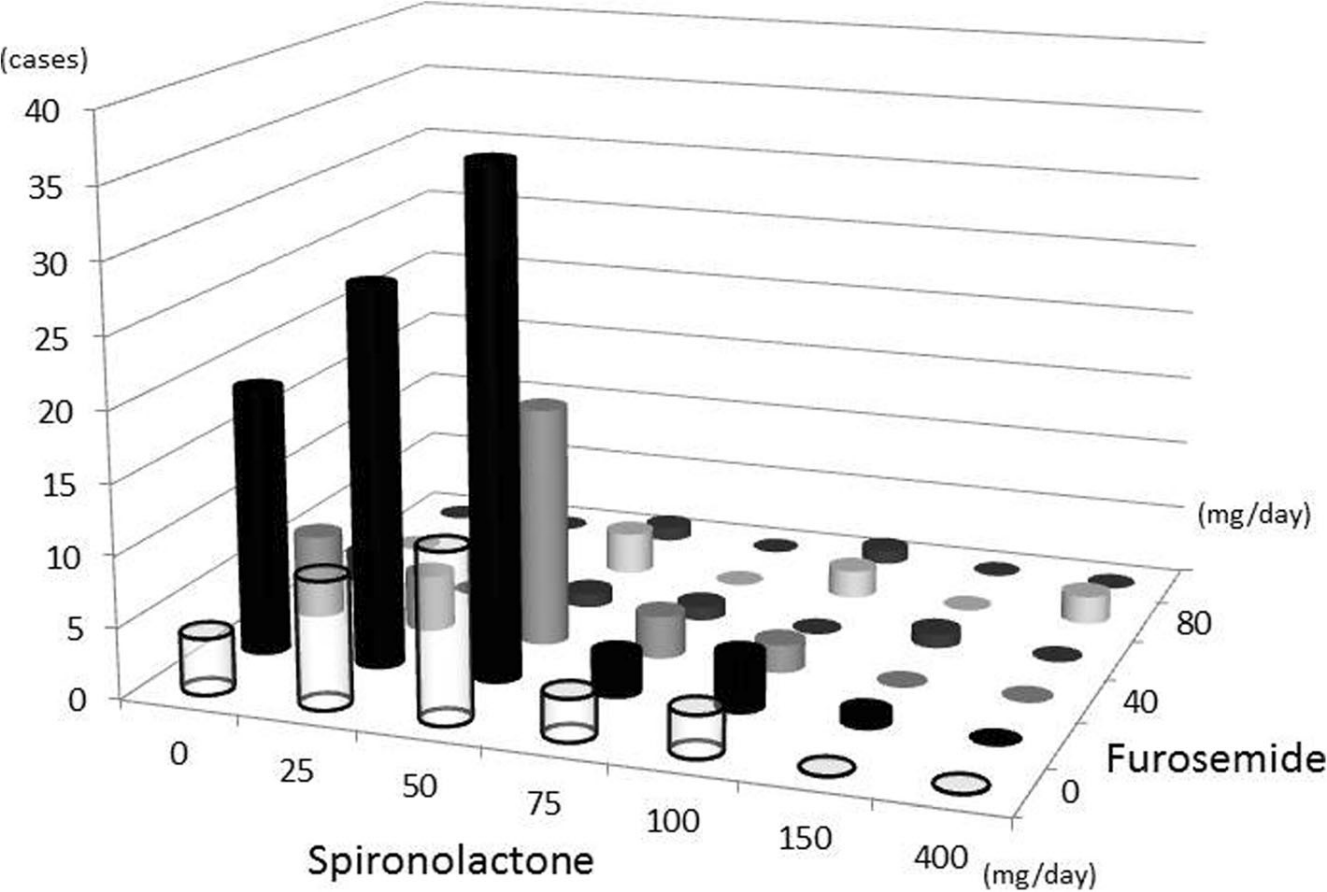
Distribution of diuretic use before tolvaptan treatment. The majority of cirrhotic patients with ascites who started tolvaptan treatment were taking 0–50 mg/day of spironolactone and 20–40 mg/day of furosemide

The distribution of diuretic use is shown in Fig. 2. The majority of cirrhotic patients with ascites who started tolvaptan treatment were taking 0–50 mg/day spironolactone and 20–40 mg/day furosemide.

### Patients who could stop treatment due to improved ascites

Of the patients who withdrew from tolvaptan treatment after < 1 year, the reasons included death (*n* = 59), liver transplantation (*n* = 22), hemodialysis or non-response to treatment (*n* = 9), transfer to another hospital (*n* = 28), and improvement (*n* = 8). Overall, nine patients stopped tolvaptan treatment (Table 2). Six cases had ALD, and patients who stopped alcohol consumption were able to successfully improve their ascites without tolvaptan treatment. However, two patients required retreatment, which was accompanied by a return to alcohol consumption. In a patient with methotrexate-related liver disease, ascites improved after discontinuing methotrexate. A patient with HBV temporarily recovered by undergoing nucleic acid analog treatment, but required tolvaptan treatment again.

**Table 2:** Patients who stopped tolvaptan treatment due to improved ascites

Age (years old)	Sex	Etiology of liver disease	Tolvaptan treatment (days)	Requirement for restarting of tolvaptan	Furosemide (mg/day)	Spironolactone (mg/day)	Modified CP score	Event
60s	Male	Alcoholic liver disease	126		80	400	11	Discontinued drinking
40s	Male	Hepatitis C virus-related liver disease+ alcoholic liver disease	1226	Yes	20	25	13	Discontinued drinking
40s	Male	Alcoholic liver disease	49	Yes	20	100	12	Discontinued drinking
70s	Male	Primary sclerosing cholangitis	335		160	100	11	Decreased protein urea
60s	Male	Hepatitis B virus-related liver disease	249	Yes	20	25	10	Treatment with nucleic acid analog
80s	Female	Hepatitis C virus-related liver disease	187		20	50	8	Disappearance of portal vein thrombosis
70s	Male	Alcoholic liver disease	293		20	25	10	Discontinued drinking
60s	Male	Alcoholic liver disease+MTX associated liver disease	186		10	25	11	Discontinued MTX
60s	Female	Alcoholic liver disease	139		10	25	12	Discontinued drinking

*CP* Child-Pugh, *MTX* methotrexate

### Comparison of laboratory data between patients who withdrew and continued tolvaptan treatment

**Fig. 3:**
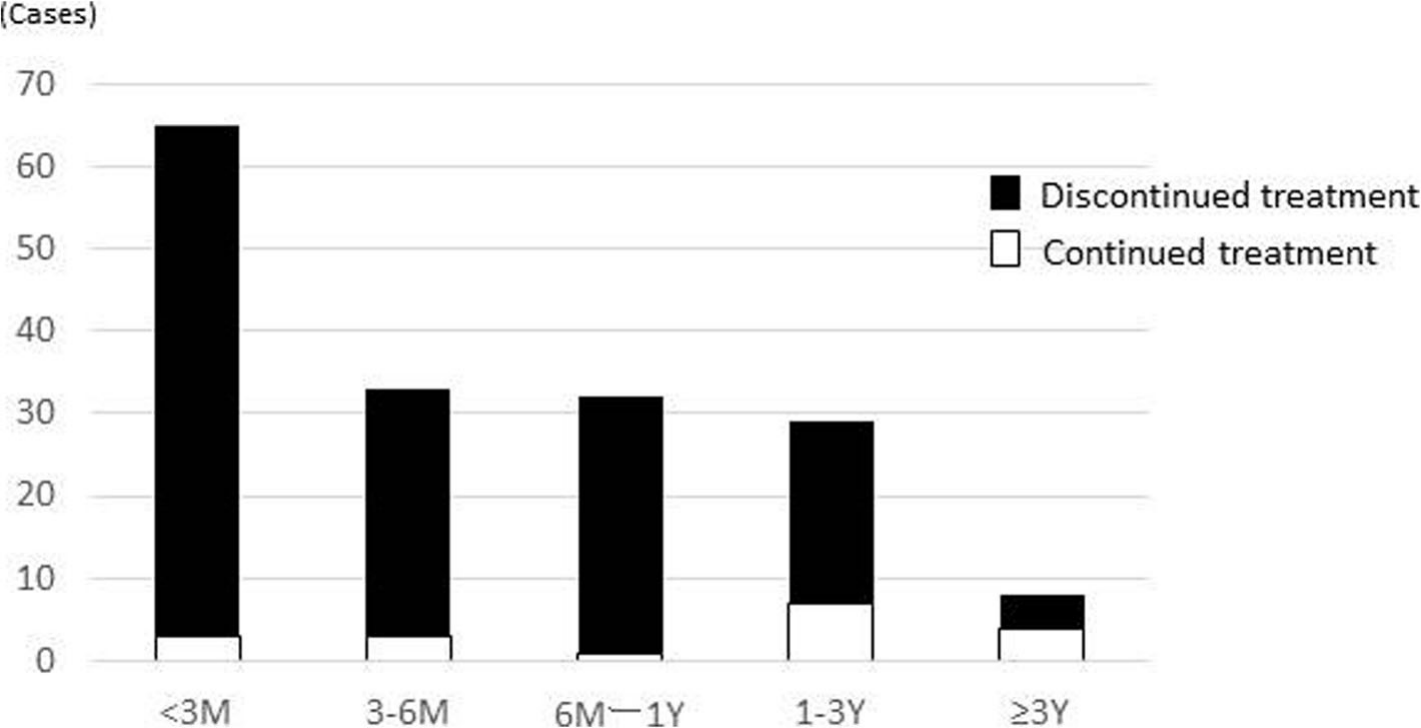
Distribution of tolvaptan treatment period. Overall, of the patients who discontinued tolvaptan treatment, 62 discontinued within 3 months, 30 in 3–6 months, and 31 in 6 months to 1 year. Only 37 patients were treated for ≥1 year. The median treatment period was 132 days

Tolvaptan was introduced to cirrhotic patients with ascites who were also taking conventional diuretics. However, almost all of the patients could not continue long-term treatment (Fig. 3). Overall, 62 patients discontinued tolvaptan within 3 months, 30 in 3–6 months, and 31 in 6 months to 1 year. Only 37 cases were treated for ≥1 year. The median treatment period was 132 days.

Patients who were treated with tolvaptan for < 1 year (*n* = 90, without improved and continued treating cases) were compared to those treated for ≥1 year (*n* = 37, Fig. 1, Table 1). Changes in body weight after 1 week were significantly greater in patients who continued treatment (− 2.0 kg [− 17.2 to + 3.4]; *p* = 0.03) than in those who discontinued treatment (− 1.1 kg [− 7.5 to + 6.2]). Consistent with the etiology of liver disease, the rate of ALD increased in such patients, who also had less gastroesophageal varices. A Denver shunt was placed in three patients who continued treatment and in only one patient who discontinued treatment. The rates of other ascites therapies including diuretic use, administration of BCAA, and CART or drainage of ascites were not significantly different between the groups.

The serum levels of albumin (p = 0.03) and sodium (*p* = 0.01) were significantly higher, whereas the level of total bilirubin (*p* < 0.01) was significantly lower in patients who continued treatment. Although the modified CP score tended to be low (mean value 11 [range: 8–13], *p* = 0.05) in patients who continued treatment, the eGFR was slightly reduced (49.5 [16.8–107.2] mL/min/1.73 m^2^ [*p* = 0.07]) in that group.

### Changes in chemical parameters from baseline, and analyses of factors predictive of continued long-term tolvaptan treatment

**Fig. 4:**
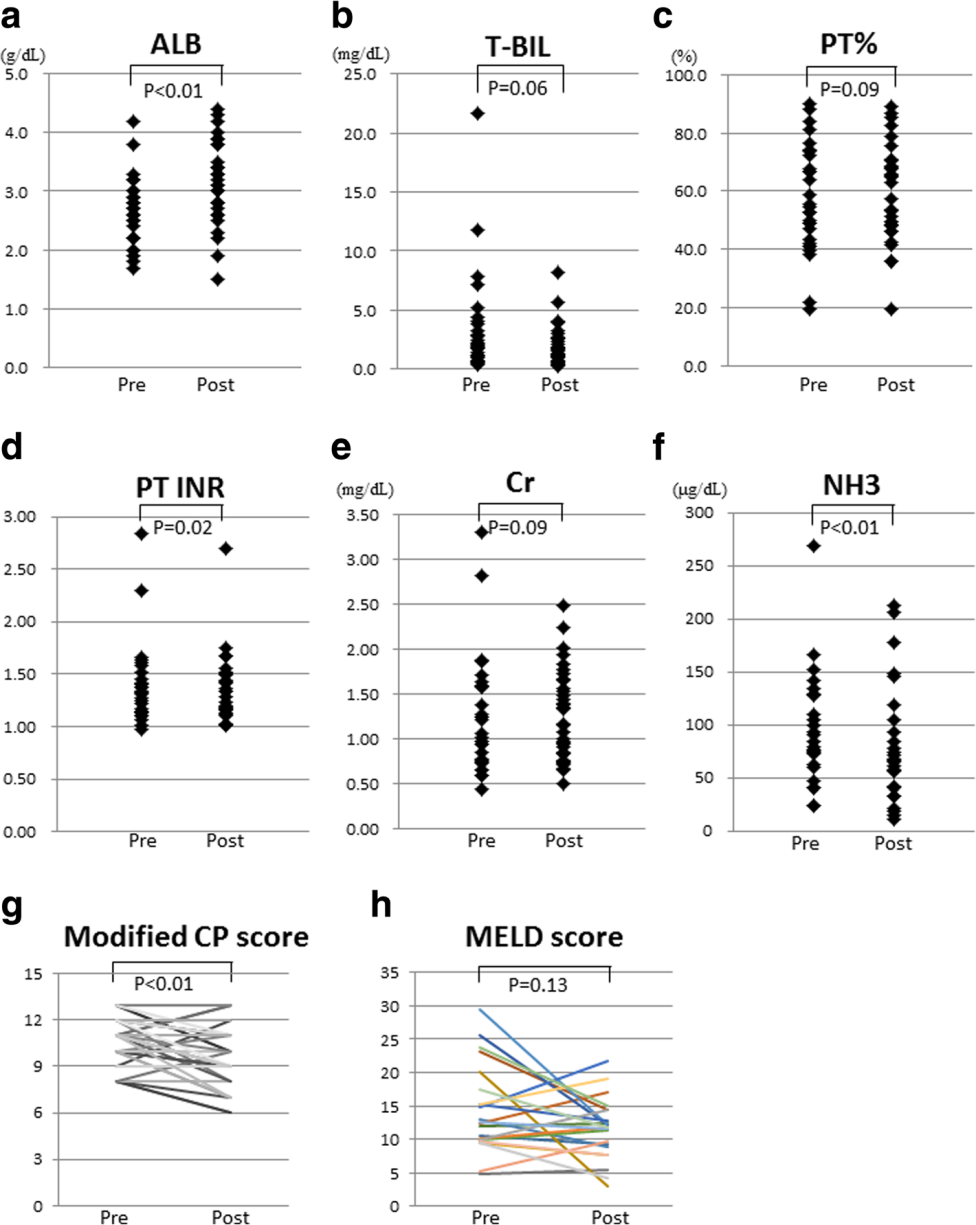
Changes in biochemical parameters in blood after 1 year of tolvaptan. **a**) Serum levels of albumin, **b**) total bilirubin, **c**) PT%, **d**) PT/INR, **e**) creatinine, **f**) ammonia, **g**) the modified CP score, and h) MELD score. Albumin levels were significantly increased (a, *p* < 0.01), whereas total bilirubin (b, *p* = 0.06), PT/INR (d, *p* = 0.02), and ammonia (f, *p* < 0.01) were reduced after 1 year of tolvaptan treatment. The modified CP score was significantly improved (g, *p* < 0.01). The MELD score was also reduced, however, it not significantly (h, *p* = 0.13). PT, prothrombin time; INR, international normalized ratio; CP, Child- Pugh; MELD, model for end-stage liver disease

To investigate whether long-term tolvaptan treatment may improve liver and renal function, patients who were treated for ≥1 year were assessed (*n* = 37, Table 3). Serum levels of albumin were significantly increased (*p* < 0.01, Fig. 4a), whereas levels of total bilirubin (*p* = 0.06, Fig. 4b) and ammonia (p < 0.01, Fig. 4f), and the PT/INR (*p* = 0.02, Fig. 4d) were significantly reduced after 1 year of tolvaptan treatment. Renal function was not affected (Fig. 4e). The modified CP score improved significantly (*p* < 0.01, Fig. 4g) and the MELD score was also reduced, but not significantly (*p* = 0.13, Fig. 4h).

**Table 3:** Changes in laboratory data from baseline, and diuretic use after 1 year of tolvaptan treatment

(*n* = 37)	Pretreatment	Post-treatment	*p*-value
Diuretic			
Furosemide dose (mg/day)	20 (0–80)	20 (0–80)	0.28
Spironolactone dose (mg/day)	50 (0–150)	37.5 (0–150)	0.50
Laboratory data			
Albumin (g/dL)	2.6 (1.7–4.2)	3.1 (1.5–4.4)	< 0.01
Total bilirubin (mg/dL)	2.0 (0.4–21.7)	1.5 (0.3–8.2)	0.01
Aspartate aminotransferase (U/L)	42 (13–171)	34 (15–701)	0.09
Alanine aminotransferase (U/L)	29 (7–144)	20 (9–255)	0.14
γ-Glutamyl transpeptidase (U/L)	48 (9–229)	40 (12–285)	0.52
Platelet count (×10^4^/μL)	7.6 (1.5–23.9)	7.7 (1.6–23.6)	0.64
Hematocrit (%)	28.6 (24.2–37.3)	32.0 (18.8–40.7)	0.06
Prothrombin time (PT%)	54.5 (19.5–90.3)	60.4 (19.4–89.4)	0.09
PT/INR	1.32 (0.98–2.84)	1.26 (1.01–2.70)	0.02
Ammonia (μg/dL)	85 (25–269)	67 (12–212)	< 0.01
α-fetoprotein (ng/mL)	4 (1–210)	3 (1–17,734)	0.79
Des-gamma-carboxy (mAU/mL)	27 (3–4994)	27 (8–40,510)	0.53
Blood urea nitrogen (mg/dL)	23.2 (6.0–63.3)	25.4 (6.0–75.2)	0.99
Creatinine (mg/dL)	1.04 (0.44–3.30)	1.16 (0.50–2.48)	0.09
eGFR (mL/min/1.73 m^2^)	49.5 (16.8–107.2)	42.9 (15.3–136.3)	0.42
Serum sodium (mEq/L)	137 (122–145)	138 (123–144)	0.63
Modified Child-Pugh (CP) score	11 (8–13)	10 (6–13)	< 0.01
MELD score	12 (2–29)	12 (3–22)	0.13

*INR* international normalized ratio, *eGFR* estimated glomerular filtration rate, *n* number of patients, *MELD* model for end-stage liver disease. Data are represented as the median (range)

To identify predictive factors associated with continued administration of tolvaptan, multivariate logistic regression analyses were used to assess changes in body weight, gastroesophageal varices, HCC, hepatic encephalopathy, eGFR, and levels of albumin, total bilirubin, aspartate aminotransferase, and sodium (Table 4). Changes in body weight and albumin levels were identified as predictive factors (odds ratio [OR]: 1.320, 95% confidence interval [CI]: 1.083–1.608, *p* = 0.006 and OR: 4.280, 95% CI: 1.136–16.127, *p* = 0.032, respectively). In addition, eGFR was weakly associated with continued administration (OR: 0.978, 95% CI: 0.958–0.999, *p* = 0.045).

**Table 4:** Predictive factors for continued administration of tolvaptan for 1 year

	Odds ratio	95% confidence interval	*p*-value
Change in BW after 1 week (kg)	1.320	1.083–1.608	0.006
Albumin (g/dL)	4.280	1.136–16.127	0.032
eGFR (mL/min/1.73 m^2^)	0.978	0.958–0.999	0.045

Changes in body weight (BW), complications including gastroesophageal varices, hepatocellular carcinoma, and hepatic encephalopathy, and lab values including estimated glomerular filtration rate (eGFR) and serum levels of albumin, total bilirubin, aspartate aminotransferase, and sodium were assessed using multivariate logistic regression analyses

## Discussion

We demonstrated that tolvaptan treatment for ≥1 year significantly increased albumin levels and decreased the PT/INR and ammonia levels. Factors associated with continued administration included body weight reduction after 1 week and better liver function.

In the current study, patients who required liver transplantation were included. Therefore, it was difficult to analyze long-term tolvaptan treatment, because the modified CP score had already attained 11 (range: 6–14). In this condition, 37 cases (22%) with a modified CP score of 11 (8–13) were observed for ≥1 year. Serum levels of albumin were significantly higher in continuously treated cases. While hematocrit values were slightly increased by improving excess water, total bilirubin and ammonia levels were decreased. Therefore, the increase in albumin level was thought to be due to improved condition. Although the MELD score did not differ significantly between the groups, the modified CP score improved significantly in the group that continued treatment (*p* < 0.01). We speculated that patients with improved ascites had increased appetite and nutritional condition, resulting in increased protein production ability.

Long-term administration of tolvaptan resulted in no significant decrease in renal function. We previously reported that liver transplant recipients maintained their renal function after 1 year of transplantation [17]. Even in chronic kidney disease, tolvaptan reduces the risk for medium-term worsening renal function [18], because tolvaptan is an aquaretic agent that removes excess water without electrolyte excretion. Therefore, renal functional loss was not exacerbated [19]. Furosemide greatly increases urination, independent of the intravascular volume, reducing renal function.

Regarding predictors associated with long-term treatment, changes in body weight were significantly greater in continued cases. Lower complication rates of esophageal varices, total bilirubin levels, and higher serum levels of albumin and sodium were predictive factors for long-term treatment. Portal vein pressure is associated with tolvaptan response [20]. A hepatic venous pressure gradient (HVPG) of 190 mmH2O or less results in a body weight reduction of 2 kg in a week. It has been suggested that ascites accumulation is caused by not only by low albumin levels but also portal hypertension.

Multiple logistic regression analyses showed that changes in body weight reduction (OR: 1.320, 95% CI: 1.083–1.608, *p* = 0.006) and albumin levels (OR: 4.280, 95% CI: 1.136–16.127, *p* = 0.032) were predictive factors for continued treatment. In addition to the baseline ability of the liver, tolvaptan response was important for continued administration. Recently, response to tolvaptan was defined as a 1.5 kg/week reduction and this value was expected to be an indicator of symptomatic improvement [21]. Body weight reduction may also be used as a predictor of long-term outcome.

By contrast, few reports have discussed the stopping of tolvaptan treatment because liver cirrhosis is irreversible and almost all patients fail to discontinue treatment. We attempted to stop treatment by improving ascites in nine patients (one patient stopped after ≥1 year treatment); however, three patients relapsed and required retreatment. These patients stopped consuming alcohol, ceased methotrexate use, and underwent nucleic acid analog treatment for HBV. These factors may have been associated with improved ascites, leading to functional improvement of the liver, even if the liver was in a cirrhotic state. In particular, patients with ALD might have a chance to recover if they stop drinking. The number of patients who successfully completed tolvaptan treatment was limited; therefore, the predictive factors were not determined. However, cases with temporarily compromised liver function and those initially engaging in treatment may wish to discontinue the drug.

A limitation of this study was that it was a single-center observational retrospective study. We could not compare patients with and without tolvaptan therapy; therefore, the direct effects of tolvaptan on albumin levels were not determined. Further analyses to determine the outcomes of long-term tolvaptan treatment are required.

## Conclusions

In conclusion, long-term treatment with tolvaptan increased serum levels of albumin and decreased ammonia levels after 1 year of treatment. Body weight reduction after 1 week was associated with a favorable outcome of tolvaptan therapy. Among patients who require radical treatment for cirrhosis and still require ascites treatment, tolvaptan administration should be maintained.

## Abbreviations

ALD: Alcoholic liver disease
BW: Body weight
CART: Cell-free and concentrated ascites reinfusion therapy
CP: Child-Pugh
eGFR: Estimated glomerular filtration rate
HBV: Hepatitis B virus
HCV: Hepatitis C virus
INR: International normalized ratio
MELD: Model for end-stage liver disease
NAFLD: Non-alcoholic fatty liver disease
PBC: Primary biliary cholangitis
